# Endoplasmic Reticulum Adaptation and Autophagic Competence Shape Response to Fluid Shear Stress in T24 Bladder Cancer Cells

**DOI:** 10.3389/fphar.2021.647350

**Published:** 2021-05-03

**Authors:** Giorgia Del Favero, Michael Zeugswetter, Endre Kiss, Doris Marko

**Affiliations:** ^1^ Department of Food Chemistry and Toxicology, Faculty of Chemistry, University of Vienna, Vienna, Austria; ^2^ Core Facility Multimodal Imaging, Faculty of Chemistry, University of Vienna, Vienna, Austria

**Keywords:** T24 bladder cancer cells, shear stress (fluid), autophagy, endoplasmic reticulum stress response, deoxynivalenol-3-sulfate, chloroquine, cytoskeleton, rapamycin

## Abstract

Accumulation of xenobiotics and waste metabolites in the urinary bladder is constantly accompanied by shear stress originating from the movement of the luminal fluids. Hence, both chemical and physical cues constantly modulate the cellular response in health and disease. In line, bladder cells have to maintain elevated mechanosensory competence together with chemical stress response adaptation potential. However, much of the molecular mechanisms sustaining this plasticity is currently unknown. Taking this as a starting point, we investigated the response of T24 urinary bladder cancer cells to shear stress comparing morphology to functional performance. T24 cells responded to the shear stress protocol (flow speed of 0.03 ml/min, 3 h) by significantly increasing their surface area. When exposed to deoxynivalenol-3-sulfate (DON-3-Sulf), bladder cells increased this response in a concentration-dependent manner (0.1–1 µM). DON-3-Sulf is a urinary metabolite of a very common food contaminant mycotoxin (deoxynivalenol, DON) and was already described to enhance proliferation of cancer cells. Incubation with DON-3-Sulf also caused the enlargement of the endoplasmic reticulum (ER), decreased the lysosomal movement, and increased the formation of actin stress fibers. Similar remodeling of the endoplasmic reticulum and area spread after shear stress were observed upon incubation with the autophagy activator rapamycin (1–100 nM). Performance of experiments in the presence of chloroquine (chloroquine, 30 μM) further contributed to shed light on the mechanistic link between adaptation to the biomechanical stimulation and ER stress response. At the molecular level, we observed that ER reshaping was linked to actin organization, with the two components mutually regulating each other. Indeed, we identified in the ER stress–cytoskeletal rearrangement an important axis defining the physical/chemical response potential of bladder cells and created a workflow for further investigation of urinary metabolites, food constituents, and contaminants, as well as for pharmacological profiling.

## Introduction

Urinary bladder cells experience a very complex extracellular environment. Fluid shear stress originates from the movement in the organ lumen and constantly modulates the cell physiological response. At the same time, bladder cells are exposed to a “chemical challenge” related to the accumulation of xenobiotics and waste metabolites in the urine. In line, bladder cells need to maintain elevated mechanosensory competence together with sophisticated response to chemicals. Not surprisingly, in the development of novel organ-on-a-chip toxicological approaches, bladder and renal cells play a paramount role (Jang et al., 2013; Kim and Takayama, 2015; Xu et al., 2015; Chang et al., 2016; Wilmer et al., 2016; Musah et al., 2017; Vriend et al., 2019; Del Favero and Kraegeloh, 2020). Mechanistically, microfluidic devices can include shear stress or exclude it in order to create a solute gradient without a physical component (Lee et al., 2016; Oleaga et al., 2016). Indeed, urinary bladder cells´ physiology is *per se* shaped by shear stress (Carattino et al., 2013), making physiological behavior a combination of response to chemical and physical stimulation. In addition to normal cell functions, more and more studies suggest that response to shear stress also plays a central role in pathological contexts, for instance, in shaping metastatic progression (Ma et al., 2017; Huang et al., 2018; Follain et al., 2019; Yan et al., 2019). Taking these as starting points, we explored the adaptation potential of T24 bladder cancer cells to shear stress and how much of the response to physical cues can be modified by pharmacological treatment or urinary-occurring xenobiotics which share in their molecular mechanism the potential to modulate autophagy.

Among food and feed contaminants, the mycotoxin deoxynivalenol (DON) can be considered as one of the most prevalent contaminants worldwide (European Food Safety Authority, 2013; Kazemi Darsanaki et al., 2015; Liu Y. et al., 2016; Li et al., 2016; Tima et al., 2016; Katrine et al., 2017). Structurally, DON is classified as a trichothecene mycotoxin, and it is produced as a secondary metabolite of *Fusarium* spp. fungi (Yoshizawa and Morooka, 1975; Kamimura et al., 1981). At the molecular level, it inhibits protein synthesis after binding to the 60 S ribosomal subunit (Pan et al., 2014; Dellafiora et al., 2017). This results in a proteostatic insult which severely hampers cell physiological functions (Pestka, 2010a; Pestka, 2010b; Katrine et al., 2017). As a consequence, incubation with DON, among others, also results in alteration of cell cytoskeletal elements (Vandenbroucke et al., 2009; Bianco et al., 2012; Pan et al., 2013). For instance, in A431 squamous carcinoma cells, incubation with DON is accompanied by a rearrangement of tubulin and a decrease of proteins mediating cell adhesion, which ultimately account for a loss of cell biomechanical compliance (Del Favero et al., 2018b). This occurs already at concentrations that are supposed to be non-cytotoxic and do not affect the total cellular protein content. Hence, the integration of biomechanical stimulation in toxicological profiling provides very sensitive readouts and contributes to elucidate the differences between the *in vitro* and *in vivo* experimental response. At the urinary level, DON is not found exclusively as a parent compound, but also metabolized. In particular, it was already detected in the form of glucuronide conjugates (Warth et al., 2012; Sarkanj et al., 2013) and more recently also as sulfate (Warth et al., 2016). This opened the question if DON metabolites, potentially accumulating in the bladder, could account for harmful effects. We previously described how sulfate metabolites of DON (DON-3-sulfate and DON-15-sulfate) could enhance proliferation of cancer cells with little or no effect on the non-transformed counterparts (Warth et al., 2016; Del Favero et al., 2018a). However, proliferation is only one of the key features of malignant cells sustaining tumor development. Particularly, response to biomechanical stimulation and altered adaption to physical stimuli often benchmark cancer progression (Broders-Bondon et al., 2018).

Building on this, we investigated the behavior of bladder carcinoma T24 cells after incubation with urinary metabolite DON-3-Sulf, comparing static incubations and shear–stress response in a microfluidic system. The selected settings were shown previously to induce cell proliferation and to alter localization profile of autophagy marker LC3 (Warth et al., 2016; Del Favero et al., 2018a). Mechanistically, we verified the hypothesis that autophagy competence and the ER stress response could tune chemical and physical integrative pathways of T24 cells. For comparison, we included in our experimental layout the pharmacological inhibition of autophagy with chloroquine (CQ) and activation with rapamycin (mammalian target of rapamycin, mTOR inhibitor (Carloni et al., 2014)). In addition, autophagy activation contributes to shape chemoresistance in bladder cancer cells (Ojha et al., 2016; Lin J.-F. et al., 2017). For this reason, pharmacological modulation of this pathway is regularly proposed in combined therapeutic protocols (Li et al., 2019), making it also of great interest to deepen the mechanism of action in the presence of biomechanical stimulation, as well as the potential crosstalk with food contaminants and/or respective metabolites.

## Materials and Methods

### Chemicals and Reagents

DON was purchased from Romer Labs (Tulln, Austria); DON-3-Sulf was synthesized in-house as previously described (Fruhmann et al., 2014; Warth et al., 2016). The purity of DON–sulfate was >95% and determined by NMR. Chloroquine was purchased from Thermo Fisher Scientific (Waltham, MA, United States). Rapamycin (Calbiochem 553211), CK-666 (SML0006), and cytochalasin D (C2618) were purchased as ready-made stock solutions from Sigma-Aldrich. Compounds were dissolved in DMSO or water, which served as respective solvent controls. If not otherwise specified, chemicals were acquired by Sigma-Aldrich Chemie GmbH (Munich, Germany).

### Cell Culture

The urinary bladder carcinoma cell line T24 (ATCC^®^ HTB4™) was purchased from ATCC. T24 cells were cultivated in McCoy’s 5a medium modified supplemented with 10% (v/v) heat-inactivated fetal bovine serum (FBS) and 1% (v/v) penicillin/streptomycin. Cell culture media and supplements were purchased from GIBCO Invitrogen (Karlsruhe, Germany), Sigma-Aldrich Chemie GmbH (Munich, Germany), Sarstedt AG and Co. (Nuembrecht, Germany), VWR International GmbH (Vienna, Austria), and Thermo Fisher Scientific GmbH (Vienna, Austria). For cell cultivation and incubations, humidified incubators at 37°C and 5% CO_2_ were used, and cells were routinely tested for the absence of *Mycoplasma* contamination.

### Live Cell Imaging

The cells were stained for live cell imaging as previously described (Del Favero et al., 2018b; Muqaku et al., 2020). Briefly, at the end of the experimental treatment (24 h), cells were incubated with the ER staining reagent Green Detection Reagent ab139481 (dil. 1:1,000) from Abcam, Cell Mask™ Deep Red Plasma membrane Stain (dil. 1:2,000), LysoTracker^®^ Red DND-99 (dil. 1:1,000), and Hoechst 33258 pentahydrate (dil. 1:1,000) or LysoSensor™ Green DND-189 (dil. 1:1,000) from Thermo Fisher Scientific for 15 min at 37°C in Live Cell Imaging Solution (GIBCO, Invitrogen). After a subsequent washing step, the stained cells were kept in Live Cell Imaging Solution and imaged within 1 h. Cells were imaged using a Zeiss LSM710 laser scanning confocal microscope (ELYRA PS.1 system) equipped with a 63X/1.2 water immersion objective (Zeiss Microscopy GmbH, Germany) or with the Lionheart FX Automated Microscope (BioTek Instruments, Winooski, VT, United States). For image analysis, the software ZEN 2012 Black Edition (Zeiss Microscopy GmbH, Germany), Gen5 (BioTek Instruments, Winooski, VT, United States), and the free software ImageJ were used as previously described (Del Favero et al., 2018b; Neuditschko et al., 2020). For the microfluidic slides, at least eight images were taken with the phase contrast, GFP (469/525 nm), and DAPI (377/447 nm) channels resulting in minimum 24 different optical fields for quantification. Every dataset resulted from the analysis of minimum three independent cell preparations (biological replicates).

### Cell Viability and Proliferation Assays

For the determination of cell viability according to the metabolic capacity, the CellTiter-Blue assay was used (Promega Corporation, Madison, WI, United States). CellTiter-Blue reagent solution was diluted according to the specification of the supplier (1:10) with pre-warmed colorless Dulbecco Modified Eagle Medium (DMEM). To allow the development of the reaction, cells were incubated in the dark at 37°C for 90 min. The fluorescence signal developing in the supernatant was measured in black plates with a photometer (*λ* = 560Ex/590 Em nm). For the determination of cell viability on the basis of cell biomass, cells were rinsed with pre-warmed D-PBS and fixed for 10 min with ice-cold EtOH (99%). Afterward, cells were stained for 5 min with Crystal Violet solution (0.1%) and rinsed four times with autoclaved dH_2_O. As the last step, cells were lysed with a mixture of 99% cold EtOH and 1% of acetic acid (10 min, shaking incubation 500 U/Min), and absorbance was measured (λ = 595 nm) with a Cytation 3 multi-mode plate reader (BioTek Instruments, Winooski, VT, United States). Every dataset resulted from the analysis of minimum three independent cell preparations, and measurements were performed in technical triplicates.

### Biomechanical Stimulation

Shear stress experiments were performed using an Ibidi microfluidic perfusion system (Ibidi, Gräfelfing, Germany) (Del Favero et al., 2018c). The Ibidi Quad Air pump system equipped with a perfusion set WHITE/10963/50 cm/ID 0.8 mm was installed in a humidified incubator maintaining 37°C and 5% CO_2_ inside. This system allows the application of the shear stress stimulation protocol to maximum four different incubation slides, making it possible to test in parallel multiple experimental conditions. The pump system was controlled using the software Ibidi-pump control v 1.5.0. 48 h Prior to the shear stress experiments, the cells were seeded at a density of 18.000 cells/200 µL in µ-Slide 0.4 Luer Collagen IV or µ-Slide 0.4 Luer ibiTreat chambers (Ibidi, Gräfelfing, Germany) and cultivated in a humidified incubator at 37°C and 5% CO_2_. Before the shear stress experiments, cells were incubated with toxins and/or pharmacological reagents for 24 h. Shear stress was applied for 3 h using a flow rate of 0.03 ml/min. These parameters were chosen on the basis of the literature reporting the response of bladder cancer cells to shear stress (Lee et al., 2018) and according to stress response sensitivity of our system.

To detect the morphological changes of the cells before and after the biomechanical stimulation, bright field images were acquired using an Olympus CKX53 Inverted Microscope. For every experiment, at least three images were acquired before (static controls) and after the biomechanical stimulation protocol (shear stress), resulting in a minimum of nine different optical fields for subsequent single-cell morphometric quantification (Neuditschko et al., 2020).

### Immunocytochemistry

Fluorescent staining of T24 cells was performed in µ-Slide eight well Collagen IV and µ-Slide eight well ibiTreat (Ibidi, Gräfelfing, Germany). Immunofluorescence localization of L1CAM, LAMP-2, Cathepsin D (CTSD), and actin was performed as previously described with a minor modification (Del Favero et al., 2020b; Gerner et al., 2020; Meier-Menches et al., 2020). Briefly, after formaldehyde fixation, cells were rinsed and permeabilized with 0.2% Triton X-100 followed by blocking with 2% donkey serum. Afterward, slides were incubated with primary antibodies at +4°C overnight (L1CAM mouse monoclonal antibody, ab 24345) or for 2 h at RT (anti–LAMP-2 mouse monoclonal antibody [H_4_B_4_]-Lysosome Marker ab25631 and Anti-Cathepsin D rabbit monoclonal antibody [EPR3057Y] ab75852, and Anti-IRE1 rabbit polyclonal antibody ab37073, all from Abcam, Cambridge, United Kingdom). After multiple washing steps, the Alexa Fluor 488 donkey anti-mouse IgG (H + L), Alexa Fluor 568 donkey anti-rabbit IgG (H + L), or Alexa Fluor 647 donkey anti-mouse IgG (H + L) (Molecular Probes-Life Technologies) were used as secondary antibodies. Actin filaments were detected with phalloidin conjugated either with Alexa Fluor 546 or with Alexa Fluor 488 (Molecular Probes-Life Technologies), following the description of the supplier. At the end of staining, the samples were mounted and sealed with Roti-Mount FluorCare with DAPI (Roth, Graz, Austria). Imaging was performed using a Zeiss LSM710 laser scanning confocal microscope (ELYRA PS.1 system) equipped with a 63X/1.4 plan-apochromat oil immersion objective (Zeiss Microscopy GmbH, Germany). Image analysis was performed with the software ZEN 2012 Black Edition (Zeiss Microscopy GmbH, Germany) according to Jarolim et al. (2017), Del Favero et al. (2018b), and Del Favero et al. (2020b) with minor adaptation. In order to allow a comparison between the experimental conditions, mean fluorescence intensity, expressed as relative fluorescence units (r.f.u.), was recorded applying constant acquisition parameters including signal/noise calibration and background correction. In order to ensure unbiased selection of the regions of interests (ROI), channels of target fluorophores were temporarily disabled, and cell nuclei (DAPI) and actin cytoskeleton were used as reference for the selection of nuclear and the cytoplasmic compartments, respectively. Measurements were performed on three cell preparations analyzing more than 40 cells randomly chosen from at least six different optical fields.

### Statistical Analysis

Data were evaluated with the software OriginPro 2018b (OriginLab Corporation, Northampton, United States). Multiple comparisons of independent samples were performed with the one-way ANOVA test followed by the Fisher’s test. Student’s *t*-tests were applied for the direct comparison of groups of data. Distributions were considered statistically different using a threshold value of 0.05.

## Results

### Effect of Deoxynivalenol-3-Sulf on the Endoplasmic Reticulum and Lysosomes of T24 Cells

**FIGURE 1:**
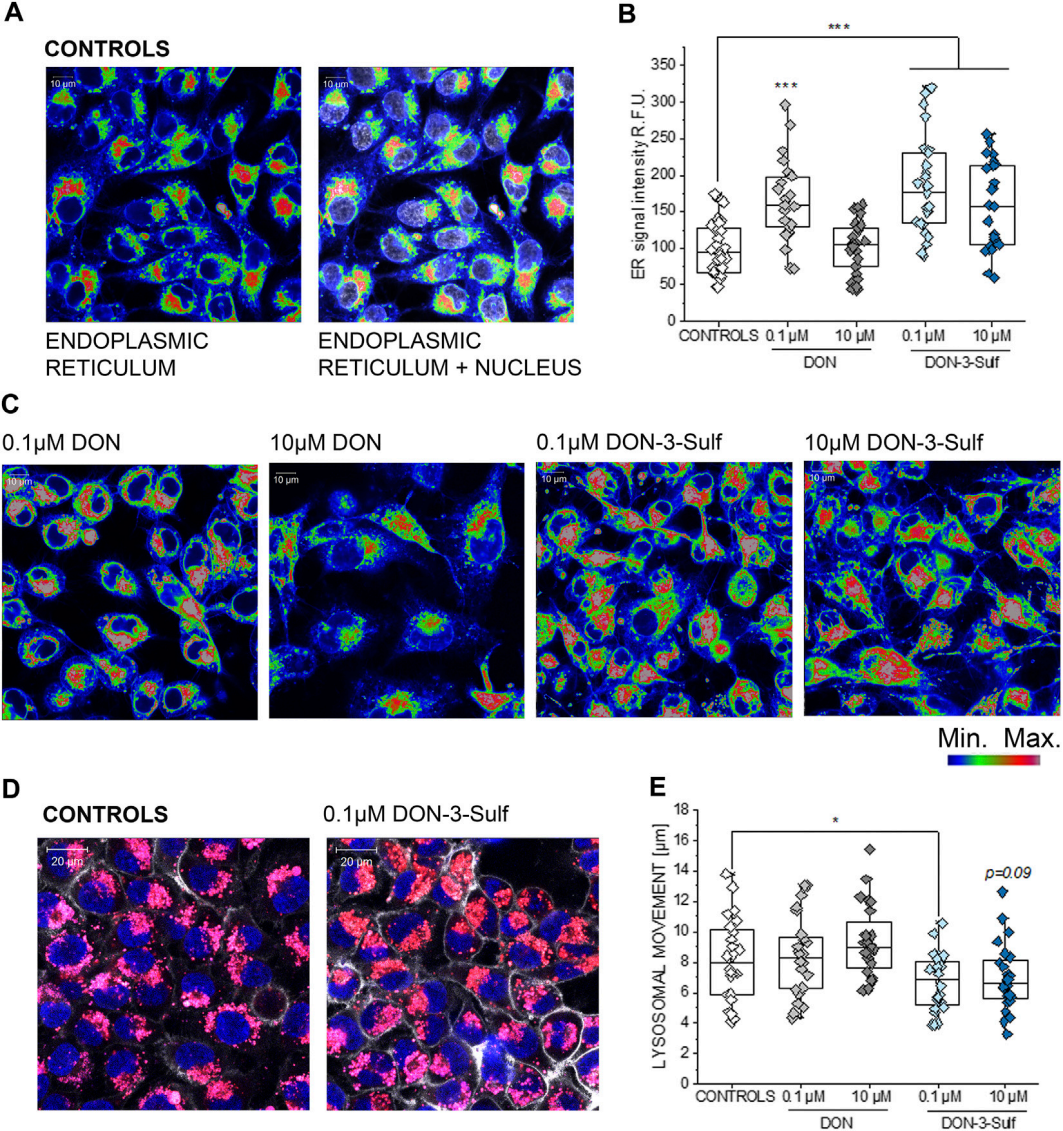
Response of the endoplasmic reticulum (ER) to the incubation with deoxynivalenol (DON) and DON-3-Sulf in T24 bladder cancer cells. **(A)** Representative image of the appearance of control cells. **(B)** Quantification of the signal intensity of the ER after 24 h incubation with DON, DON-3-Sulf, and controls. Data are obtained by the quantification of *n* > 25 region of interest (relative fluorescent units; r. f.u.). *** indicates significant difference in comparison to controls at the Student’s *t*-test (*p* < 0.001; scale bar: 10 µm). **(C)** Appearance of the ER after incubation with DON and DON-3-Sulf. **(D)** Evaluation of lysosomes after 24 h incubation with deoxynivalenol (DON) and DON-3-Sulf in T24 bladder cancer cells. Representative images of the appearance of control cells and cells incubated with DON-3-Sulf. Lysosomes are depicted in red, cell nuclei in blue, and the cell membrane in white. **(E)** Quantification of the lysosomal motility. Data are expressed as lysosomal movement across 20 time frames after manual tracking of five particles/cell from *n* = 24 randomly chosen cells derived from three independent preparations (scale bar: 20 µm).

Exposure of T24 cells to DON-3-Sulf was previously reported to be only moderately cytotoxic and to rather stimulate cancer cell proliferation (Warth et al., 2016). Additional experiments performed on colon cancer HT-29 cells led to postulate that DON-sulfate metabolites could exert their biological activity by modulating autophagy (Del Favero et al., 2018a). This line of interpretation implied a prolonged sub-toxic proteostatic insult, ultimately modifying protein turnover and cell growth profile. To verify if this hypothesis could find confirmation in the response of bladder cells, we decided to investigate the effect of DON-3-Sulf on the endoplasmic reticulum (ER). Indeed, the ER stress response can integrate with autophagy in case of a proteostatic insult or protein misfolding (unfolded protein response, UPR) (Cybulsky, 2017; Smith and Wilkinson, 2017). In line, we observed that incubation of T24 cells with DON-3-Sulf enlarged the ER in T24 bladder cancer cells (Figure 1). This was accompanied by an increase of the fluorescence signal originating from the staining of the organelle. The parent compound DON induced a similar response, but only in the concentration 0.1 μM (Figures 1B,C). In addition, we also observed that 24 h incubation with DON-3-Sulf decreased the motility of the lysosomes and favored their accumulation in the perinuclear region (Figures 1D,E).

### Effect of Deoxynivalenol-3-Sulf and Chloroquine on Cell Viability and Autophagy Markers

**FIGURE 2:**
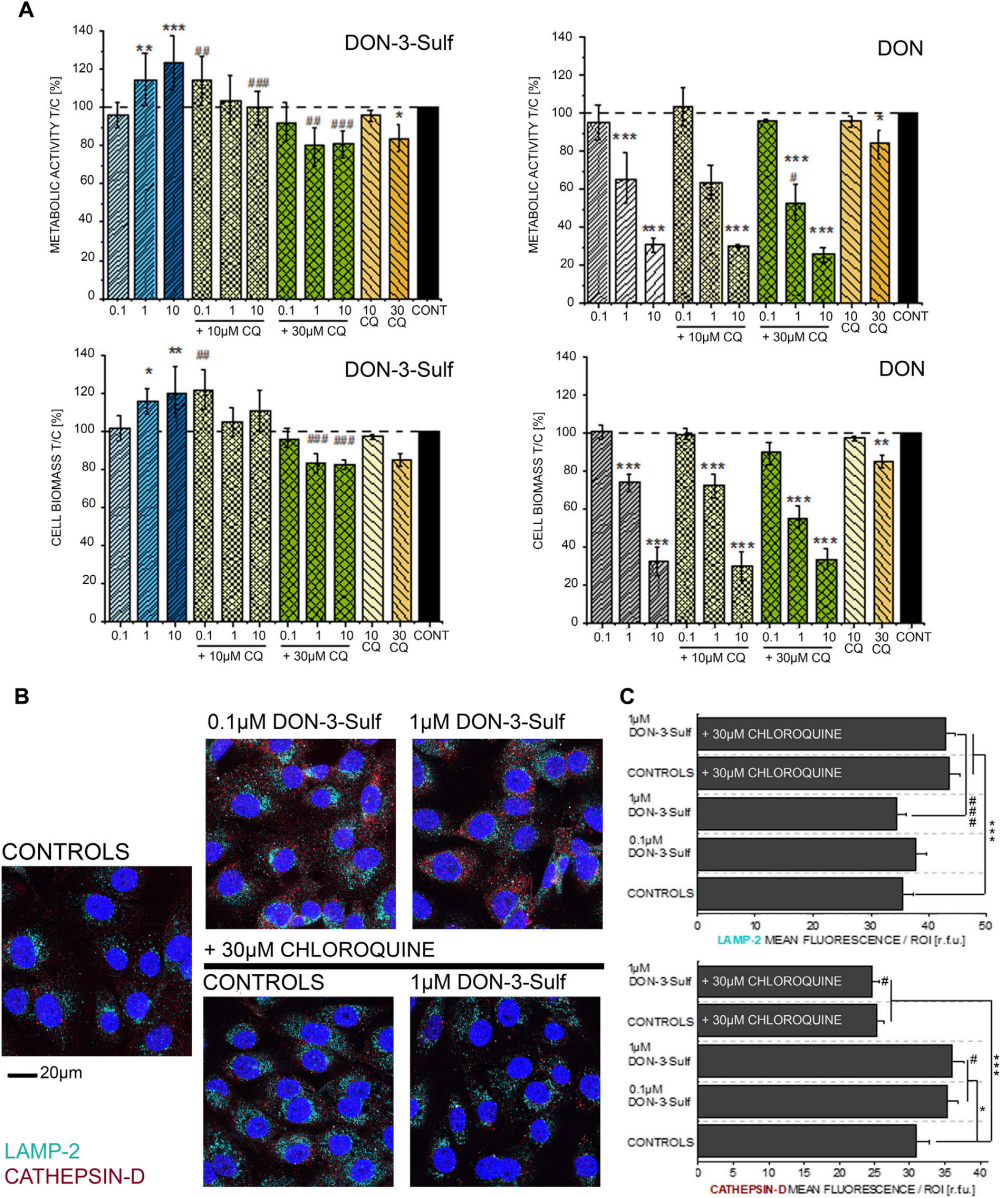
**(A)** Effect of DON-3-Sulf and DON on T24 cell viability measured as metabolic activity (Alamar blue assay) and cell biomass (crystal violet assay). * indicates significant difference in comparison to controls and # difference between incubation conditions with and without CQ (#*: *p* ≤ 0.05; ##**: *p* ≤ 0.01; ###***: *p* ≤ 0.001). **(B)** Appearance of T24 cells after immunofluorescence localization of LAMP-2 (light blue) and cathepsin D (red). Cell nuclei are depicted in blue (DAPI; scale bar: 20 µm). **(C)** Quantification of the fluorescence signal of LAMP-2 and cathepsin D expressed as mean/ROI (relative fluorescent units; r. f.u.); n > 60 cells from three different preparations. * indicates significant difference in comparison to controls and # difference between incubation conditions with and without CQ (#*: *p* ≤ 0.05; ###***: *p* ≤ 0.001) at the Student’s *t*-test.

Since DON-3-Sulf appeared to impact two major components of the protein turnover apparatus like the endoplasmic reticulum and lysosomes, we decided to verify to what extent the activity of the urinary metabolite could be modulated by the presence of a classical autophagy inhibitor, namely, chloroquine (CQ) (Redmann et al., 2017; Lin Y.-C. et al., 2017). For the first insight in the response profile of T24 bladder cells to autophagy modulators, we measured the cytotoxic and pro-proliferative potential of DON-3-Sulf and compared the response in the presence or absence of CQ. With this experimental layout, we observed how the pro-proliferative effect of DON-3-Sulf could be abolished by co-incubation with CQ (Figure 2A). For comparison, the parent mycotoxin DON was also tested. Incubation with DON decreased the viability of T24 cells in a concentration-dependent manner, and this effect was not modulated by co-incubation with CQ (Figure 2A). In addition, immunofluorescence analysis of two classical lysosomal–autophagosomal markers like lysosome-associated membrane protein-2 (LAMP-2) (Eskelinen et al., 2002; Vion et al., 2017) and the lysosomal endopeptidase cathepsin D (Benes et al., 2008) was also performed. Incubation with CQ clearly changed the intracellular pattern profile of LAMP-2 in comparison to controls; increase of the fluorescence signal was localized, especially in the perinuclear region (Figures 2B,C). On the other hand, incubation with DON-3-Sulf increased the signal of cathepsin D, and this response was abolished by the co-incubation with 30 µM CQ (Figures 2B,C).

### Effect of Deoxynivalenol-3-Sulf on T24 Cell Response to Shear Stress

**FIGURE 3:**
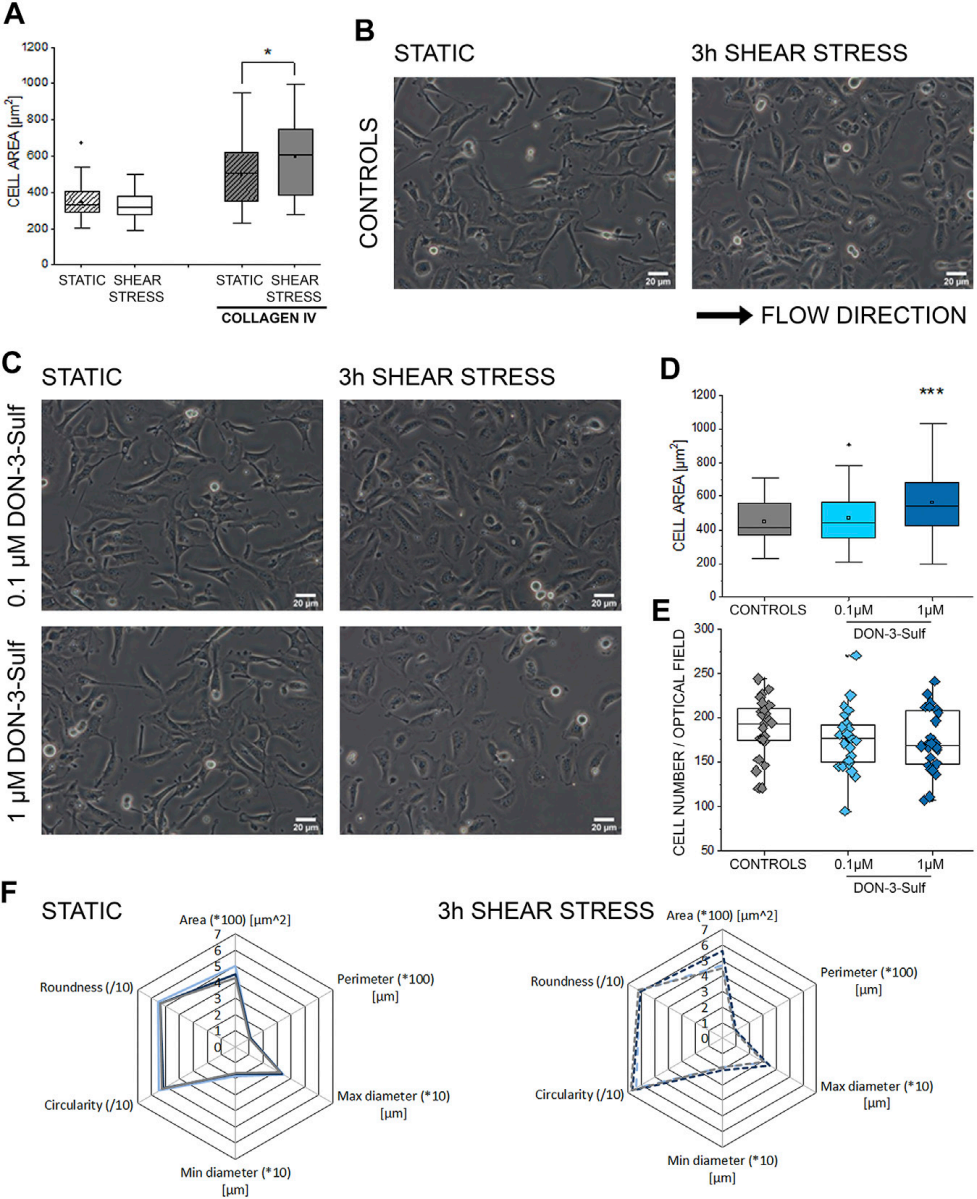
Response of T24 to shear stress stimulation. **(A)** Quantification of area spread after biomechanical stimulation. **(B)** Bright field appearance of T24 cells in static conditions and after 3 h shear stress on collagen IV extracellular matrix. **(C)** Bright field appearance of T24 cells after incubation with DON-3-Sulf 0.1–1 µM. **(D)** Area distribution of cells after shear stress stimulation protocol. **(E)** The average cell number per optical field. **(F)** Multiparametric morphological evaluation of cells cultivated in static conditions and after biomechanical stimulation. Controls in gray, DON-3-Sulf in light blue (0.1 µM) and dark blue (1 µM). Morphometric data are mean of *n* = 75 cells randomly selected and quantified. * indicates significant difference in comparison to controls at the Student’s *t*-test (*: *p* ≤ 0.05; **: *p* ≤ 0.01; ***: *p* ≤ 0.001; scale bar: 20 µm).

Activation of autophagy sustains invasiveness of bladder cancer cells (Tong et al., 2019). Likewise, the expression of cathepsin D is related to cancer cell migratory potential and motility (Zhang C. et al., 2018; Zhang M. et al., 2018). Along this line, we pursued the idea that DON-3-Sulf could have an impact on the T24 cells’ response to physical cues. Shear stress is an essential component of the physiological environment of bladder cells; hence the response profile to this stimulus represents a central functional feature for this cell type. As the first step, we observed that when cultivated on collagen IV-coated slides, T24 bladder cells could modify their shape within 3 h of stimulation (Figures 3A,B). This was visible in the morphologic adaptation toward a more spread phenotype and a significant increase in the surface area. Upon incubation with DON-3-Sulf in static conditions (24 h, Figure 3C), cells were indistinguishable from untreated ones. However, upon application of shear stress, cells preincubated with the sulfate metabolite spread more broadly than controls (Figures 3C,D). This effect was not related to variation in cell density (Figure 3E). Other parameters of the multiple morphometric evaluation like circularity, roundness, diameter, perimeter, and minor/major axes remained unaffected (Figure 3F).

### Effect of Deoxynivalenol-3-Sulf and Chloroquine on T24 Cells Response to Shear Stress

**FIGURE 4:**
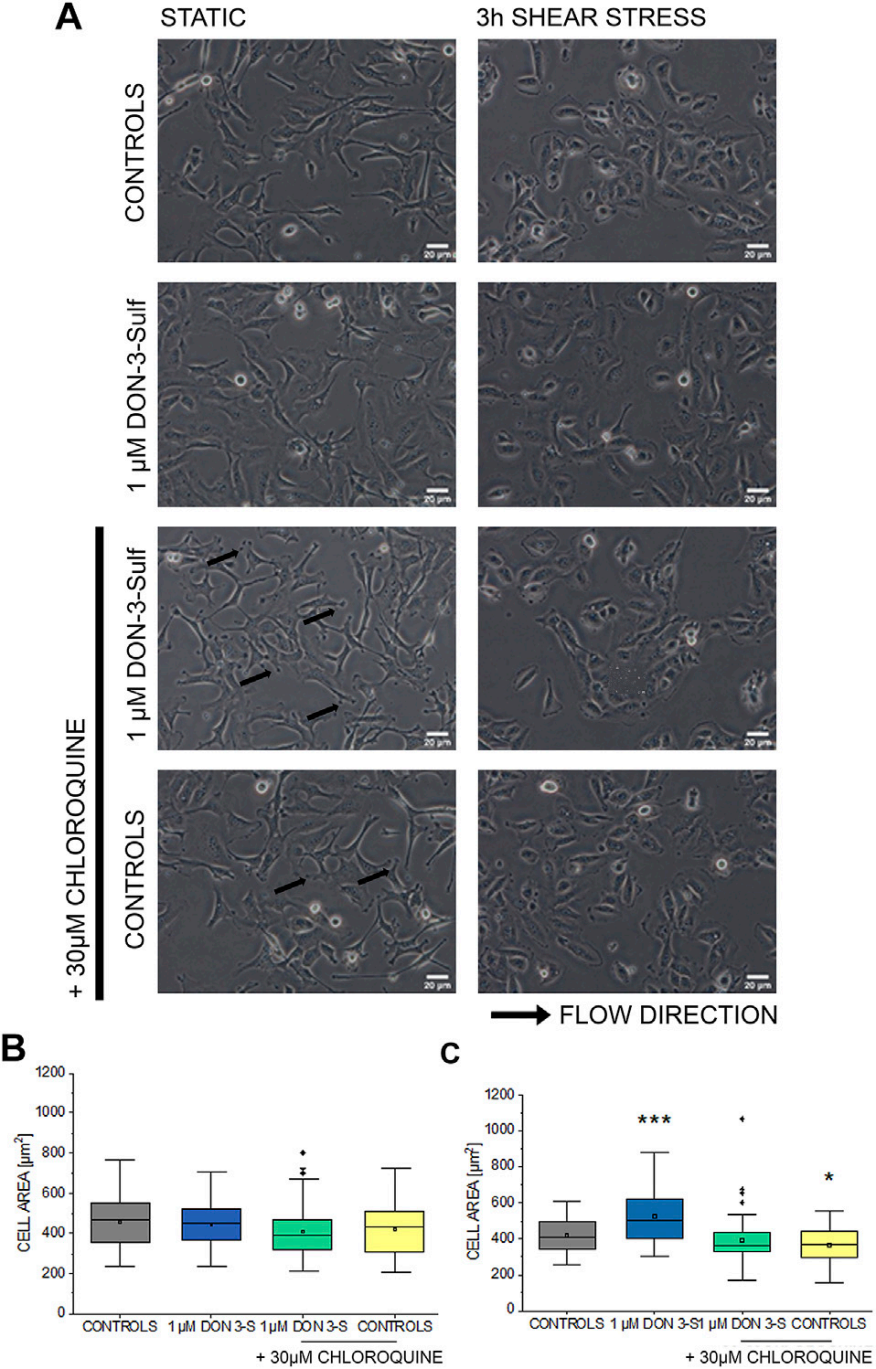
Response of T24 to shear stress stimulation. **(A)** Bright field appearance of T24 cells after incubation with 1 µM DON-3-Sulf and 30 µM chloroquine (CQ). **(B)** Quantification of cell area in static conditions and **(C)** after biomechanical stimulation. Morphometric data are mean of *n* = 45 cells randomly quantified from three independent cell preparations. * indicates significant difference in comparison to controls at the Student’s *t*-test (*: *p* ≤ 0.05; ***: *p* ≤ 0.001; scale bar: 20 µm).

**FIGURE 5:**
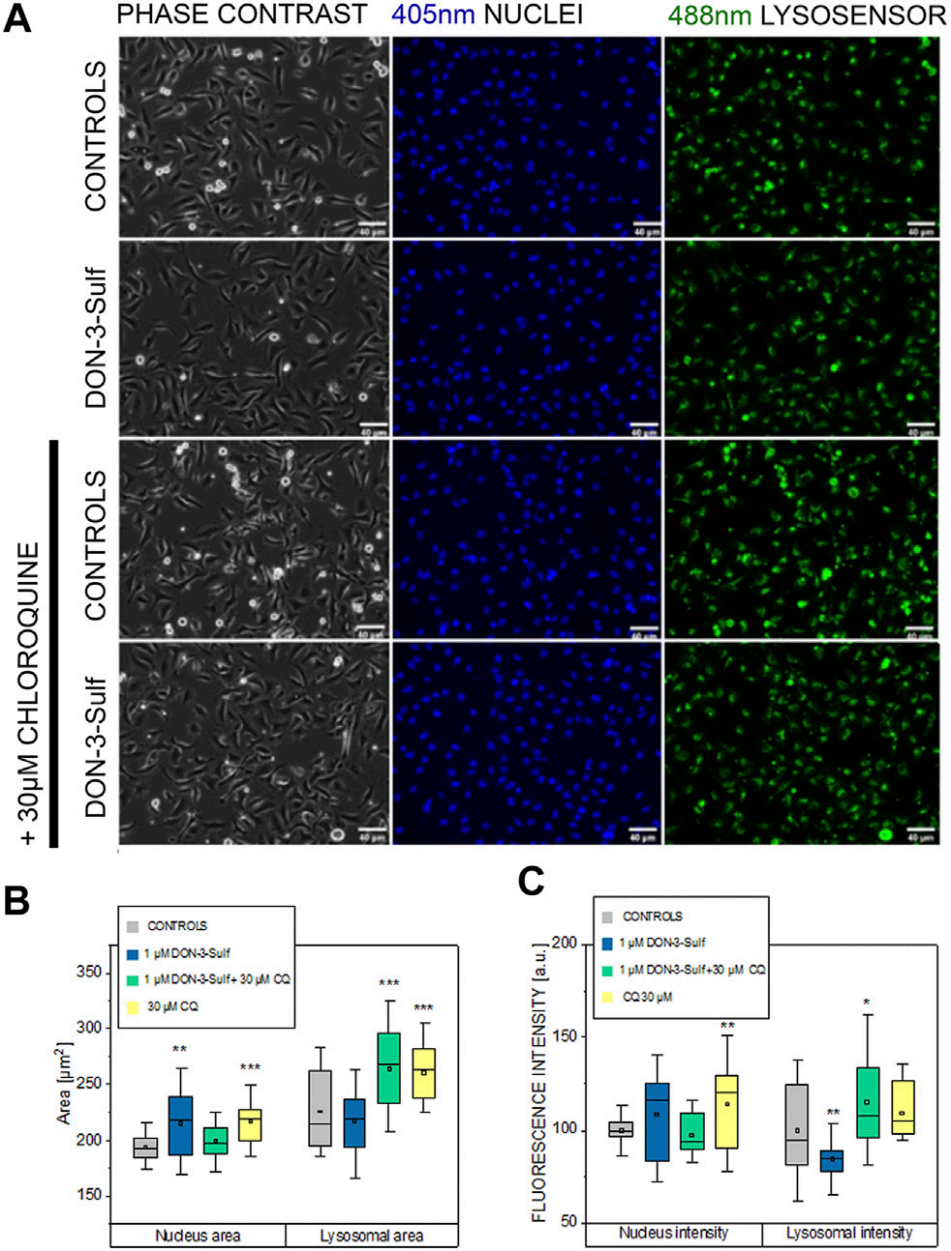
Response of T24 to shear stress stimulation. **(A)** Phase contrast and fluorescence images after staining with Hoechst (nuclei 405 nm, blue) and Lysosensor (lysosomes 488 nm, green) after incubation with 1 µM DON-3-Sulf and/or 30 µM chloroquine (CQ). **(B)** and **(C)** Image analysis of nuclear and lysosomes area and fluorescence intensity (arbitrary units). Data are mean of *n* = 24 randomly chosen fields of view imaged in the microfluidic channels. * indicates significant difference in comparison to controls at the Student’s *t*-test (*: *p* ≤ 0.05; **: *p* ≤ 0.01; ***: *p* ≤ 0.001; scale bar: 40 µm).

Pursuing the hypothesis that morphometric adaption induced by incubation with DON-3-Sulf could follow the same molecular effectors described for proliferation, we repeated the experiments in the presence of CQ (30 µM). Also, in this case, when cells were kept in static vessels, no difference in the cell area could be observed (Figures 4A,B). However, in the presence of CQ, cells developed digit-like protrusions suggestive of increased formation of focal adhesions (Figure 4A, black arrows). Consistent to the dose–response experiments, upon application of shear stress, cells incubated with DON-3-Sulf spread more than the control cells. Co-incubation with CQ proved effective in blocking this response (Figures 4A,C). In addition, in order to start shedding light on the functional status of the cells after application of biomechanical stimulation, we included in our evaluation live cell imaging experiments and stained cell nuclei with Hoechst and lysosomes with LysoSensor™ Green DND-189. In this way, we also verified if the incubation with DON-3-Sulf and CQ could impact morphology and intracellular distribution of cell nuclei and lysosomes. The cell surface area covered by the lysosomes was significantly increased by the presence of 30 µM CQ (Figures 5A,B). As for the lysosome signal, this was reduced upon incubation with DON-3-Sulf (Figures 5A,C). According to the specification of the supplier, the dye LysoSensor™ can be used to measure the pH of acidic organelles like the lysosomes. Accordingly, since the dye becomes more fluorescent in acidic environment, signal intensity variation upon incubation with DON-3-Sulf and DON-3-Sulf/CQ possibly reflected the variation of lysosomal pH. Moreover, we observed that incubation with either DON-3-Sulf or CQ increased the nuclear area (Figures 5A,B). This response was antagonized by the co-incubation of the two compounds. Intensity of the nuclear staining (Figure 5C) was increased by incubation with CQ, displaying a profile which retraced the changes in the nuclear area (Figures 5B,C).

### Effect of Deoxynivalenol-3-Sulf and Chloroquine on T24 Cells Cytoskeleton

**FIGURE 6:**
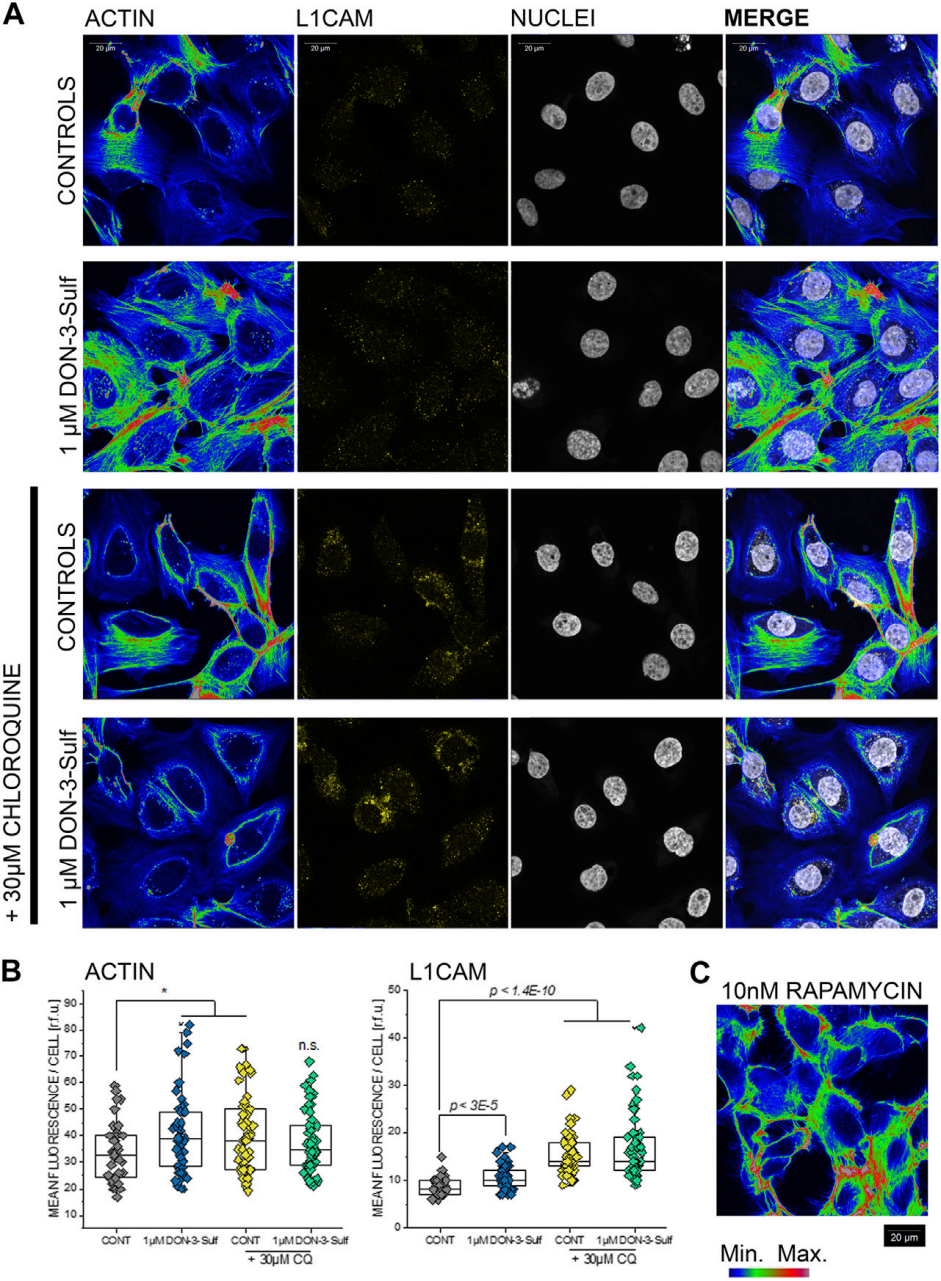
Actin cytoskeleton and L1 cell adhesion molecule (L1CAM) in T24 cells. **(A)** Representative appearance of cells in control conditions and after 24 h incubation with 1 µM DON-3-Sulf and/or 30 µM chloroquine (CQ). **(B)** Mean fluorescence signal intensity/cell expressed as relative fluorescence units (r.f.u.). **(C)** Image representative of the appearance of actin cytoskeleton after incubation with rapamycin (10 nM, 24 h). Data result from the quantification of *n* > 40 cells *p* values refer to the comparison to controls at the Student’s *t*-test (*: *p* ≤ 0.05; scale bar: 20 µm).

Since the cell nucleus behaves as a sensitive cellular mechanosensor (Isermann and Lammerding, 2013; Enyedi et al., 2016), we were intrigued by the observation that both, DON-3-Sulf and CQ, modify the nuclear area as single compounds and that the combination of the two compounds abolished this response. In this respect, nucleoskeleton and cytoskeleton are bridging into each other (Khatau et al., 2012; Lanzicher et al., 2015) and together contribute to cell biomechanical compliance. In line, we decided to verify the effect of DON-3-Sulf and chloroquine on actin cytoskeleton of T24 cells. Intriguingly, we observed that both molecules incubated as single compounds induced the formation of actin stress fibers (Figures 6A,B). A similar effect could be obtained with autophagy activator rapamycin (Kim et al., 2002) (10 nM, Figure 6C). In agreement with the response to the shear stress protocol, the combination of DON-3-Sulf and CQ reduced the formation of stress fibers. In addition to actin, we verified the immunolocalization of another molecule involved in cell adhesion and mechanotransduction, namely, L1CAM (Samatov et al., 2016). Indeed, incubation with DON-3-Sulf increased the expression of L1CAM (Figures 6A,B). However, this response was enhanced by co-incubation with CQ (Figures 6A,B), possibly following the re-localization pattern of actin toward the cell periphery.

### Effect of Rapamycin on T24 Cell Response to Shear Stress

**FIGURE 7:**
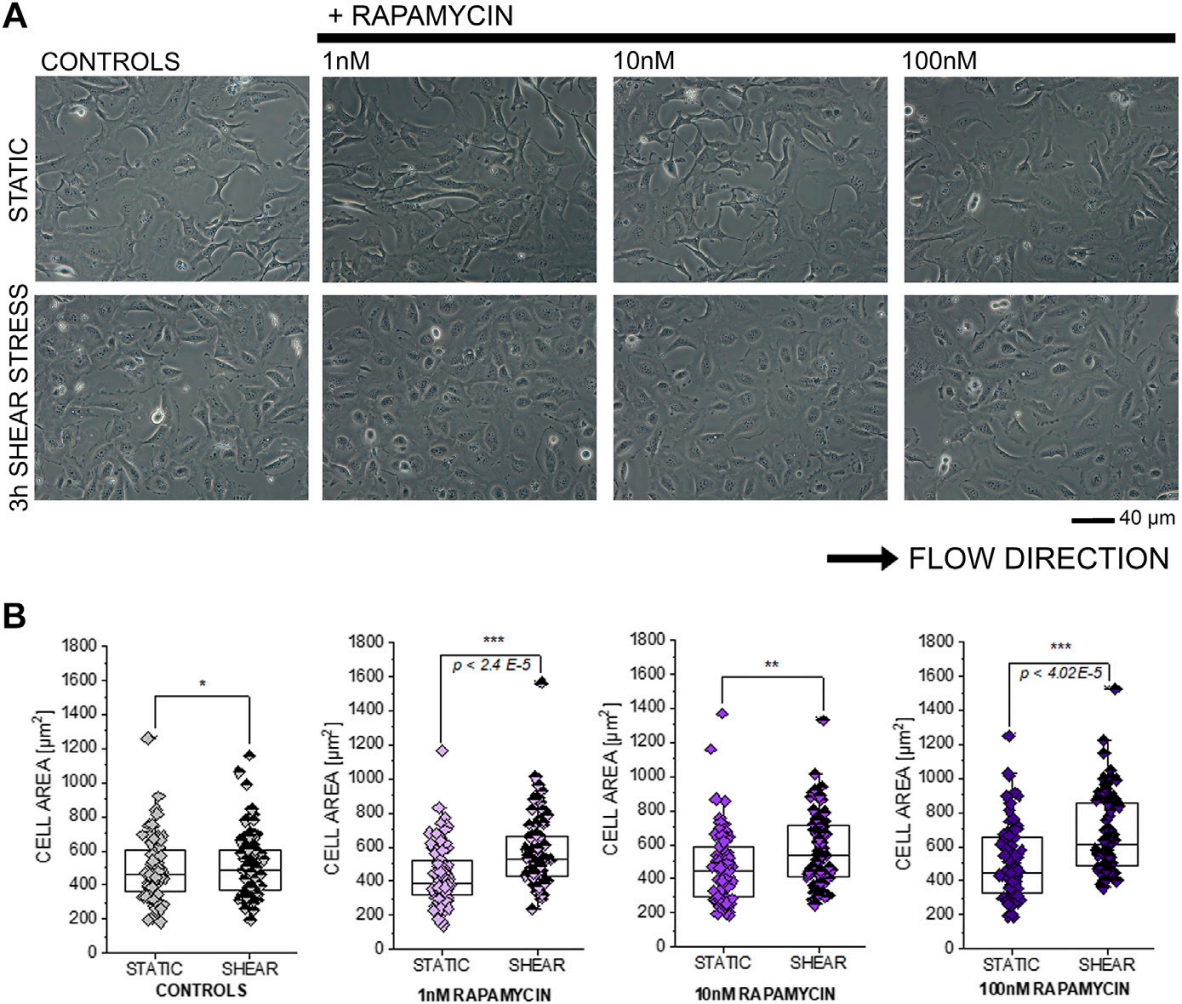
Response of T24 to shear stress stimulation. **(A)** Bright field appearance of T24 cells after incubation with rapamycin (1–100 nM). **(B)** Quantification of cell area in static conditions and after biomechanical stimulation. Morphometric data are mean of *n* = 70 cells randomly quantified from three to four independent cell preparations. * indicates significant difference in comparison to controls at the Student’s *t*-test (*: *p* ≤ 0.05; **: *p* ≤ 0.01 ***: *p* ≤ 0.001; scale bar: 40 µm).

In order to further explore the connection between compounds modulating autophagy and functional response of T24 cells, additional shear stress experiments were performed after exposure to autophagy activator rapamycin (Kim et al., 2002; Zhou and Huang, 2011; Tuloup-Minguez et al., 2013; Vion et al., 2017; du Toit et al., 2018). In line with previous results, incubation with rapamycin 1–100 nM in static conditions did not modify the appearance of T24 cells (Figures 7A,B). On the other hand, application of 3 h shear stress stimulation protocol resulted in a consistent and significant increase of the surface area spread (Figures 7A,B).

### Cytoskeleton Manipulation and ER Morphometric Profiling

**FIGURE 8:**
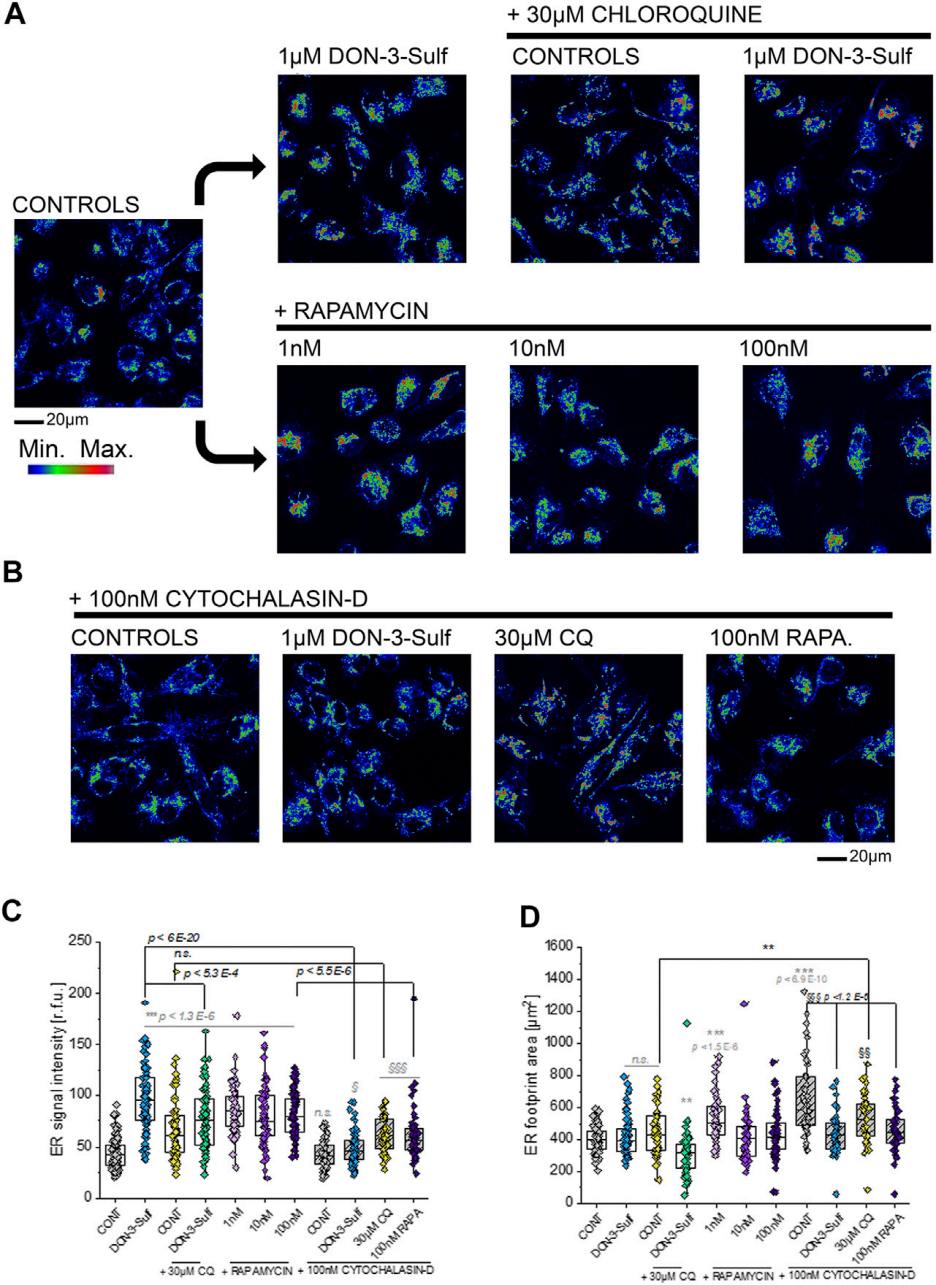
Morphometric profiling of the endoplasmic reticulum (ER). **(A)** Representative appearance of cells in control conditions and after 24 h incubation with 1 µM DON-3-Sulf and/or 30 µM chloroquine (CQ) or 1–100 nM rapamycin (RAPA.). **(B)** Representative appearance of cells after 24 h incubation with 100 nM cytochalasin D and 1 µM DON-3-Sulf, 30 µM chloroquine, or 100 nM rapamycin. **(C)** Quantification of the signal intensity of the ER expressed as relative fluorescent units (r.f.u.). **(D)** Quantification of the average ER footprint area/cell. For every condition, data result from the quantification of *n* > 50 ROI/cells. Gray symbols: * indicates significant difference in comparison to controls at the Student’s *t*-test (*: *p* ≤ 0.05; **: *p* ≤ 0.01 ***: *p* ≤ 0.001). Black symbols: * indicates significant difference between incubations with or without cytochalasin D at the Student’s *t*-test (*: *p* ≤ 0.05; **: *p* ≤ 0.01; ***: *p* ≤ 0.001). § indicates significant difference in comparison to cytochalasin D controls at the Student’s *t*-test (§: *p* ≤ 0.05; §§: *p* ≤ 0.01 §§§: *p* ≤ 0.001; scale bar: 20 µm).

**FIGURE 9:**
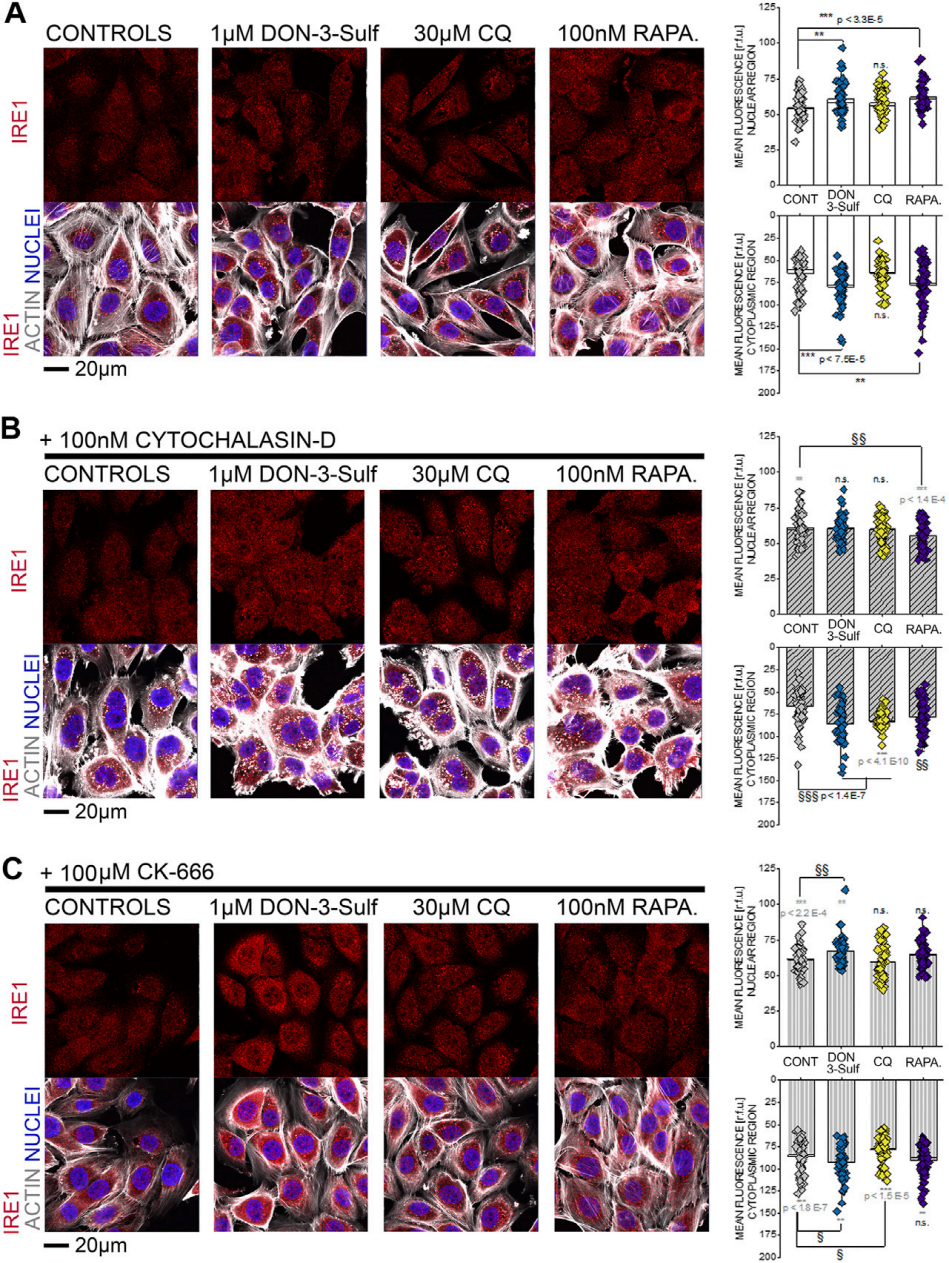
T24 cells after immunofluorescence localization of IRE1 (red). Actin cytoskeleton is depicted in white and nuclei in blue. **(A)** Representative appearance of cells in control conditions and after 24 h incubation with 1 µM DON-3-Sulf, 30 µM chloroquine (CQ), or 100 nM rapamycin (RAPA.). **(B)** Representative appearance of cells after 24 h incubation with 100 nM cytochalasin D and 1 µM DON-3-Sulf, 30 µM chloroquine, or 100 nM rapamycin (RAPA.). **(C)** Representative appearance of cells after 24 h incubation with 100 µM CK-666 and 1 µM DON-3-Sulf, 30 µM chloroquine, or 100 nM rapamycin (RAPA.). Quantification of the signal intensity of IRE1 in the nuclear and cytoplasmic regions expressed as relative fluorescent units (r.f.u.). For every condition, data result from the quantification of *n* > 50 ROI. * indicates significant difference in comparison to controls (black) or reference conditions without cytochalasin D and CK-666 (gray) at the Student’s *t*-test (*: *p* ≤ 0.05; **: *p* ≤ 0.01; ***: *p* ≤ 0.001). § indicates significant difference in comparison to cytochalasin D/CK-666 controls at the Student’s *t*-test (§: *p* ≤ 0.05; §§: *p* ≤ 0.01 §§§: *p* ≤ 0.001; scale bar: 20 µm).

Since the T24 response to shear stress did not seem to retrace exclusively autophagy activation–inhibition profile, we pursued the hypothesis that biomechanical compliance could follow the connection between the cytoskeleton and ER. For the treatments tested in the shear stress experiments, the ER signal was quantified for intensity and for distribution within the cells (footprint area). Upon incubation with 1 µM DON-3-Sulf or rapamycin (1–100 nM), the ER signal increased significantly in T24 cells. This could be observed also for cells incubated with CQ, albeit less pronounced (Figures 8A,C). Following the same pattern of the actin rearrangement and shear stress response, co-incubation of DON-3-Sulf and CQ reduced significantly the area spread of the ER (Figures 8A,D). Moreover, we performed additional experiments to verify how cytoskeletal manipulation can potentially affect the ER appearance and if this could change the response profile to DON-3-Sulf, CQ, and rapamycin. Addition of the cytoskeleton modifying agent cytochalasin D (100 nM, Figure 8B (Casella et al., 1981; Wakatsuki et al., 2001; Lanzicher et al., 2015)) resulted in a significant increase of the area spread of the ER with no effect on the mean signal intensity (Figures 8C,D). Both DON-3-Sulf and rapamycin reduced this effect with a similar activity profile. Albeit active, the incubation with CQ resulted less efficient in this respect. Intriguingly, comparing the response profile with and without cytochalasin D, actin modulation significantly reduced the ER signal increase triggered by DON-3-Sulf and rapamycin (Figures 8B,C), and no effect could be observed for CQ. On the other hand, incubation with CQ plus cytochalasin D significantly increased the ER area in comparison to CQ alone, which could not be observed for either DON-3-Sulf or rapamycin (Figures 8B–D). Since cytoskeletal manipulation proved effective in tuning the ER response to DON-3-Sulf, CQ, and rapamycin, additional experiments were performed to explore the connection with IRE1 (inositol-requiring protein 1). IRE1 serves as ER stress signal and activator of the unfolded protein response (UPR, (Adams et al., 2019; Rainbolt and Frydman, 2020). Since in the ER patterning we observed rearrangement from the cell periphery to the nuclear/perinuclear region, signal intensity was quantified in proximity to the nuclear area, as well as in the cytoplasmic compartment, taking cell nuclei (DAPI) and actin as references. Incubation with DON-3-Sulf and rapamycin significantly increased the signal of IRE1. No effect could be observed upon incubation with CQ (Figure 9A). Incubation with cytochalasin D moderately increased the IRE1 signal in the nuclear region in comparison to controls. Concomitant presence of rapamycin reduced this response (Figure 9B). In the cytoplasmic compartment, diffuse actin remodeling was accompanied by significant increase of IRE1 signal for all the combinatory treatments (DON-3-Sulf, CQ, and RAPA.; Figure 9B) in comparison to cytochalasin D alone. Considering the direct link between IRE1 and the cytoskeleton (Urra et al., 2018), we also explored the effect of a second cytoskeletal modulating agent, namely, CK-666. CK-666 targets the Arp2/3 complex, thus affecting the formation of actin filaments (Hetrick et al., 2013). In T24 cells, treatment with CK-666 decreased the intensity of actin staining and reduced the formation of stress fibers. In parallel, the IRE1 signal increased in both nuclear and cytoplasmic regions in comparison to solvent controls (Figure 9C). DON-3-Sulf further enhanced this readout, which was reduced by the co-incubation with CQ (cytoplasmic compartment) and remained unaffected in the presence of rapamycin (Figure 9C).

## Discussion

The ER stress response and autophagy contribute to support cell survival after experiencing physical and chemical challenges (Xu et al., 2005; King et al., 2011; Navarro-Yepes et al., 2014; Jacob et al., 2017; Smith and Wilkinson, 2017). These adaptive responses are essential for the bladder where cells are constantly exposed to a very complex physical–chemical environment. From the oncological perspective, considering the complexity of these pathways, it is not surprising that activation of the ER stress response or autophagy is described to have either positive or detrimental effects, largely depending on the cell type and disease stage (Ma and Hendershot, 2004; Galluzzi et al., 2015; Galluzzi et al., 2017; Wen and Klionsky, 2020; Chen and Cubillos-Ruiz, 2021). In addition to the open questions on the biochemical pathways triggered by ER stress and autophagy, consequences on cell biophysical properties and morphometric adaptation routes are also emerging (Dupont and Codogno, 2016; Huang et al., 2017; Boukhalfa et al., 2020; Yang et al., 2020). In this study, we started to shed light on the correlation patterns linking ER remodeling, cytoskeletal adaption, and functional response to shear stress in T24 bladder cancer cells. In our experimental conditions, morphological adaption measured after shear stress protocol mirrored with impressive reproducibility the structural shaping of the ER, in concerted response with the actin cytoskeleton remodeling.

For the DON-3-sulfate, we initially postulated that it could accumulate in the lysosomes as previously described for other sulfate metabolites (Andreadi et al., 2014) and trigger at that point a proteostatic insult and/or altered protein homeostasis (Del Favero et al., 2018a). In line, incubation with DON-3-Sulf significantly changed the appearance of the ER and increased the immunolocalization of cathepsin D (Figures 1, 2B,C), suggesting an increased activation/need for intracellular proteases or protein turnover. This was accompanied by a significant decrease of the lysosomal movement (Figures 1D,E) and of the signal of the LysoSensor dye (Figure 5C). At the same time, the area covered by lysosomes, respective of the cell surface, remained constant (Figure 5B); this readout was independently confirmed by a consistent signal of the lysosomal membrane protein (LAMP-2) (Figures 2B,C). Hence, the variation of the LysoSensor signal upon incubation with DON-3-Sulf (Figure 5) more likely reflected a change in the pH of the organelles, which would be compatible with the accumulation of negatively charged sulfates into acidic compartments of the lysosomes. On the other hand, CQ is known to inhibit autophagy *via* reduction of the autophagosome–lysosome fusion, albeit having no effect on intra-lysosomal acidity (Mauthe et al., 2018). In agreement, we could observe a significant increase of the intracellular surface area covered by lysosomes in all the experimental conditions that included the use of CQ and no effect on signal intensity (acidity) in cells incubated with CQ alone (Figures 5B,C). Similarly, incubation with CQ was accompanied by a significant increase of the immunolocalization of LAMP-2 (Figures 2B,C).

A tight link between autophagic competence and cell biomechanical compliance is becoming evident in several cell models (Dupont and Codogno, 2016). Particularly, it was previously described how starvation-induced autophagy could increase motility and invasiveness of bladder cancer cells (Tong et al., 2019). Defective autophagy, like in the case of LAMP-2 deficiency (Danon disease), is associated with increased stiffness and reduced stress response adaptation potential in fibroblasts (Del Favero et al., 2020a). Similarly, autophagy is central in determining endothelial cell response to shear stress as physiologically needed due to the physical stimulation of the blood in the vascular lumen (Vion et al., 2017). However, physical cues are diverse in the body, and response to biomechanical stimulation *in vitro* appears to be cell type–specific retracing, to some extent, the functional ancestry of the progenitor tissue *in vivo* (Huang et al., 2018; Del Favero and Kraegeloh, 2020). Bladder cells responded to shear stress stimulation already in control conditions, and 3 h protocol was sufficient to shape their morphology (Figures 3A,B). This readout was dependent on the functionalization of the extracellular matrix, making it evident that the response of the T24 cells results from the combination of physical (shear stress) and chemical (matrix functionalization) stimuli. Along this line, the preincubation with DON-3-Sulf resulted in a concentration-dependent increase of the cell spread (Figure 3). At the concentration of 1 µM, DON-3-Sulf also increased cell metabolic competence/biomass (Figure 2). However, in the microfluidic slides, the cell number remained constant (Figure 3E), and also considering the consistency of the morphometric parameters measured in static conditions (Figure 3F), a response merely driven by variation in cell growth seemed unlikely. To support the idea that the effect of DON-3-Sulf on cell area spreading potential was not an unspecific readout, the variation of this parameter was not accompanied by changes in the cell adhesion profile: no differences could be observed in cell circularity and roundness as it could be expected in the case of detachment from the ECM (Sen and Kumar, 2009). Intriguingly, a similar response profile with increased area spread could be obtained in T24 cells upon incubation with autophagy activator rapamycin (Figure 7) and also with the incubation with CQ (Figure 4). In all, these data suggested that biomechanical response capacity of T24 could benefit of molecular mechanisms not involving exclusively autophagy activation or inhibition, but rather another level of stress management capacity.

In T24 cells response to shear stress, ER morphology and cytoskeletal remodeling provided a coherent pattern. From a structural perspective, like the cytoskeleton, also the ER is able to adapt its shape within minutes upon stimulation (Muqaku et al., 2020). Similarly, it was previously demonstrated that endothelial cells respond to atherosusceptible shear stimulation by enlarging the ER and that this event corresponds also to maximal monocyte recruitment under flow (Bailey et al., 2017). According to our data, ER reshaping potential of T24 cells paired the biomechanical compliance (Figures 1–3–7). Indeed, the ER is tightly connected with several elements of the cytoskeleton, making it plausible that physical cues and ER enlargement could mutually influence each other. For example, filamin A is deputed to the coordination of three-dimensional actin networks and ensuring the intracellular spread of the ER (Lynch et al., 2011). More recently, the UPR sensor IRE-1α was described as a direct binding partner of filamin A clearly supporting the interplay between ER stress and cytoskeleton in the definition of cell motility (Urra et al., 2018). Similarly, intermediate filaments of vimentin are essential in fibroblasts for the regulation of the ER intracellular localization (Lynch et al., 2013), and also the non-muscle myosin IIB can serve as ER stress–dependent binding partner for IRE-1α (He et al., 2012). Along this line, the morphological changes in the ER caused by DON-3-Sulf, CQ, and rapamycin (Figures 1, 8) were in agreement with the cytoskeleton adaption observed for actin and IRE1 immunolocalization (Figures 6, 9). On the other side, the actin cytoskeletal rearrangement upon incubation with cytochalasin D reflected in a change in the area spread of the ER (Figure 8) and modified the IRE1 signal increase profile (Figure 9). Co-incubation with DON-3-Sulf or rapamycin partially reduced the response to cytochalasin D (Figures 8, 9), possibly affecting the cytoskeletal turnover *via* activation of autophagy and/or increased protein recycling (Ulbricht et al., 2013). Modification of actin branching *via* CK-666 also tuned the response profile of IRE1 (Figure 9C); in this case, the cytoskeletal rearrangement was less marked than for cytochalasin D, and IRE1 signal increase could be measured in both nuclear and cytoplasmic regions. To some extent, this response was sensitive to the incubation with DON-3-Sulf and CQ (Figure 9C). Coherent with the interpretation that ER–cytoskeletal adaptation could contribute to define cells’ structural compliance, we could also measure a significant increase of the nuclear area after the shear stress protocol for cells incubated with DON-3-Sulf and CQ (Figure 5B). This response was in agreement with the behavior of actin stress fibers (Figures 6A,B) and with the ER footprint profile (Figure 8). Indeed, within the cells, structural elements are tightly connected; for instance, mutations of nuclear lamin A/C reflect also in cytoskeletal disorganization (Lanzicher et al., 2015), and *vice versa*, actin polymerization status mirrors in nuclear stiffness (Liu L. et al., 2016; Liu et al., 2019).

In addition to the mechanical changes sustaining the reorganization of the ER and actin cytoskeleton, metabolic turnover of structural proteins upon modulation of autophagy could also account for altered sensitivity of T24 to shear stress. It was previously described that autophagy regulates the turnover of the focal adhesion proteins, ultimately affecting cell migration and shape (Kenific et al., 2016). Likewise, the effect of DON-3-Sulf and CQ was not limited to actin cytoskeleton, but expanded also to another protein mediating cell adhesion and binding like L1CAM (Samatov et al., 2016). L1CAM is found to be particularly expressed in highly invasive and metastatic tumors (Raveh et al., 2009), and in its physiological turnover, L1CAM is ubiquitinated and transported into the lysosomes for degradation (Schäfer et al., 2010). In accordance with the effects of DON-3-Sulf on lysosomal motility (Figure 1) and on the protease cathepsin D (Figure 2), it is possible to hypothesize that these events could reflect in modified protein turnover and that this regulatory mechanism could extend to multiple cytoskeletal elements. In good agreement, block of the lysosomal–autophagosomal fusion induced by CQ (Mauthe et al., 2018) (Figure 5B) possibly accounted for the bulk increase of L1CAM measured in other experimental conditions. Of note, L1CAM presented particular localization in the perinuclear region (Figures 6A,B), as for the lysosomal accumulation observed for LAMP-2 (Figures 2B,C).

## Conclusion

Taken together, our study describes how xenobiotics altering lysosomal function and autophagy competence could affect bladder cells’ biomechanical compliance. At the molecular level, at least two major routes could be identified. On the one side, the effects downstream from the physiological turnover of the cytoskeletal proteins. This includes degradation in the lysosomal compartment and *de novo* biosynthesis in the ER. Moreover, the movement and the physical constraints deriving from the ER enlargement and its structural connection with the cytoskeleton seem to account for additional mechanical stress absorbing capacity. Indeed, we could observe how ER regulation is tightly related to cytoskeletal dynamics and how this retraces cell compliance to physical stimuli, at least for short-term (3 h) adaptation. Overall, the comparison between experiments performed in static conditions and in the presence of shear stress provided an important angle for the comprehension of mechanisms regulating the ER stress response and morphometric adaptation in T24 bladder cells and represents a solid basis for further toxicological profiling of pharmaceuticals, contaminants, and metabolites at the urinary level.

## Data Availability

The raw data supporting the conclusions of this article will be made available by the authors, without undue reservation.
